# Outcomes of Fluoroscopy-Free Percutaneous Intrafocal Pinning for Adult Distal Radius Fractures in a Resource-Limited Setting in South Kivu, Democratic Republic of the Congo

**DOI:** 10.7759/cureus.103801

**Published:** 2026-02-17

**Authors:** Rodrigue Mupenda Mwenibamba, Daniel S Nteranya, Christian B Wabene, Alexandre N Nakashenyi, Eben Ezer Genda, Fabrice Kibukila, Didier M Kasilembo, Desire A Munyali, Severin M Kavatsura, Zacharie T Kibendelwa, Uwonda Akinja, Tshimbila Kabangu

**Affiliations:** 1 Surgery/Orthopedics, Official University of Bukavu, University Clinics of Bukavu, Bukavu, COD; 2 Surgery/Neurosurgery, Official University of Bukavu, University Clinics of Bukavu, Bukavu, COD; 3 Surgery/Cardiothoracic and Vascular Surgery, Official University of Bukavu, University Clinics of Bukavu, Bukavu, COD; 4 Surgery/General Surgery, Official University of Bukavu, University Clinics of Bukavu, Bukavu, COD; 5 Research, Faculty of Medicine, University of Burundi, Bujumbura, BDI; 6 Surgery, Official University of Bukavu, University Clinics of Bukavu, Bukavu, COD; 7 Surgery/Pediatric Surgery, Official University of Bukavu, University Clinics of Bukavu, Bukavu, COD; 8 Pharmacology, Official University of Bukavu, Bukavu, COD; 9 Internal Medicine, University of Kisangani, Kisangani, COD; 10 Surgery, University of Mbuji-Mayi, Mbuji-Mayi, COD; 11 Surgery, University of Kisangani, Kisangani, COD

**Keywords:** distal radius fracture, eastern drc, fluoroscopy-free, low-resource settings, percutaneous pinning

## Abstract

Purpose: To assess the anatomical, radiological, and functional outcomes of fluoroscopy-free percutaneous intrafocal pinning for distal radius fractures in adults in resource-constrained settings of South Kivu, Democratic Republic of Congo.

Methods: In a prospective, multicenter, interventional study, 101 adults with distal radius fractures were treated from January 2018 to December 2023 across four hospitals. Fractures were reduced using external maneuvers and stabilized with three-pin percutaneous intrafocal pinning without fluoroscopy. Outcomes were evaluated using the Grumillier Criteria and modified Castaing classification, with a minimum six-month follow-up. Data analysis with R software (R Foundation for Statistical Computing, Vienna, Austria) included descriptive statistics, principal component analysis, and factorial analysis.

Results: Patients had a mean age of 36.41 ± 12.61 years (range: 18-70). Castaing type I fractures comprised 92.08% of cases, with types 2 and 4 each at 2.97%. Consolidation occurred at a mean of 46.82 ± 4.67 days. Complications included superficial infections (three cases), callus formation (three cases), pin migration (two cases), algodystrophy (one case), twisted pin (one case), radial sensory branch injury (one case), and thumb extensor tendon injury (one case). At a mean follow-up of 27.27 ± 11.85 months, subjective outcomes were very good in 84.16% and good in 9.90%; objective outcomes were very good in 77.23% and good in 16.83%; and radiological outcomes were very good in 75.25% and good in 19.80%. Longer rehabilitation correlated with poorer scores, while extended hospitalization was associated with worse objective outcomes. Socio-professional reintegration was reduced with poor objective scores. Age and gender had no impact on outcomes.

Conclusion: Fluoroscopy-free percutaneous intrafocal pinning is a safe and effective technique for managing distal radius fractures in low-resource settings. It delivers excellent anatomical, radiological, and functional results.

## Introduction

Distal radius fractures are among the most common injuries seen in emergency departments [1,2] and consume considerable healthcare resources [3]. Their management is challenging due to marked anatomical and clinical variability and the absence of a universally superior treatment method [4,5]. Current guidelines recommend surgical treatment for young, active patients and for fractures that, after closed reduction, still show >3 mm radial shortening, >10° dorsal tilt, or >2 mm intra-articular step-off [6,7]. In higher-demand patients meeting these criteria, surgery yields better radiological and functional outcomes than conservative management [8]. The two most common surgical options are percutaneous K-wire fixation and volar locking plate (VLP) osteosynthesis. Both techniques provide satisfactory long-term results; VLP offers faster short-term recovery and superior anatomical restoration, whereas functional outcomes become comparable after one year [9]. Intrafocal (Kapandji) pinning, first described in 1987 [10], is a minimally invasive and cost-effective K-wire technique that provides three-point buttressing inside the fracture site, prevents secondary displacement, and eliminates the need for prolonged immobilization [11]. It is traditionally performed under fluoroscopic control to ensure accurate reduction and safe implant positioning. However, intraoperative radiation exposure, even with modern mini C-arms, carries documented risks of malignancy, chronic radiation dermatitis, and cataracts for patients and surgical staff [12-14]. In many low- and middle-income countries, particularly in sub-Saharan Africa, fluoroscopy remains unavailable in most trauma centers [15]. Consequently, distal radius fractures are frequently treated non-operatively or with “blind” Kapandji-type pinning. The Democratic Republic of Congo is no exception. We therefore conducted a single-arm prospective interventional cohort without a comparator to evaluate the anatomical, radiological, and functional outcomes of fluoroscopy-free percutaneous intrafocal (Kapandji) pinning in adults with distal radius fractures in order to determine its safety and effectiveness in resource-constrained environments.

## Materials and methods

Study design

This is a single-arm prospective interventional multicenter cohort study without a comparator that analyzed 101 consecutive cases of distal radius fractures in adults treated with fluoroscopy-free percutaneous intrafocal pinning, conducted from January 1, 2018, to December 31, 2023.

Patient recruitment

**Figure 1:**
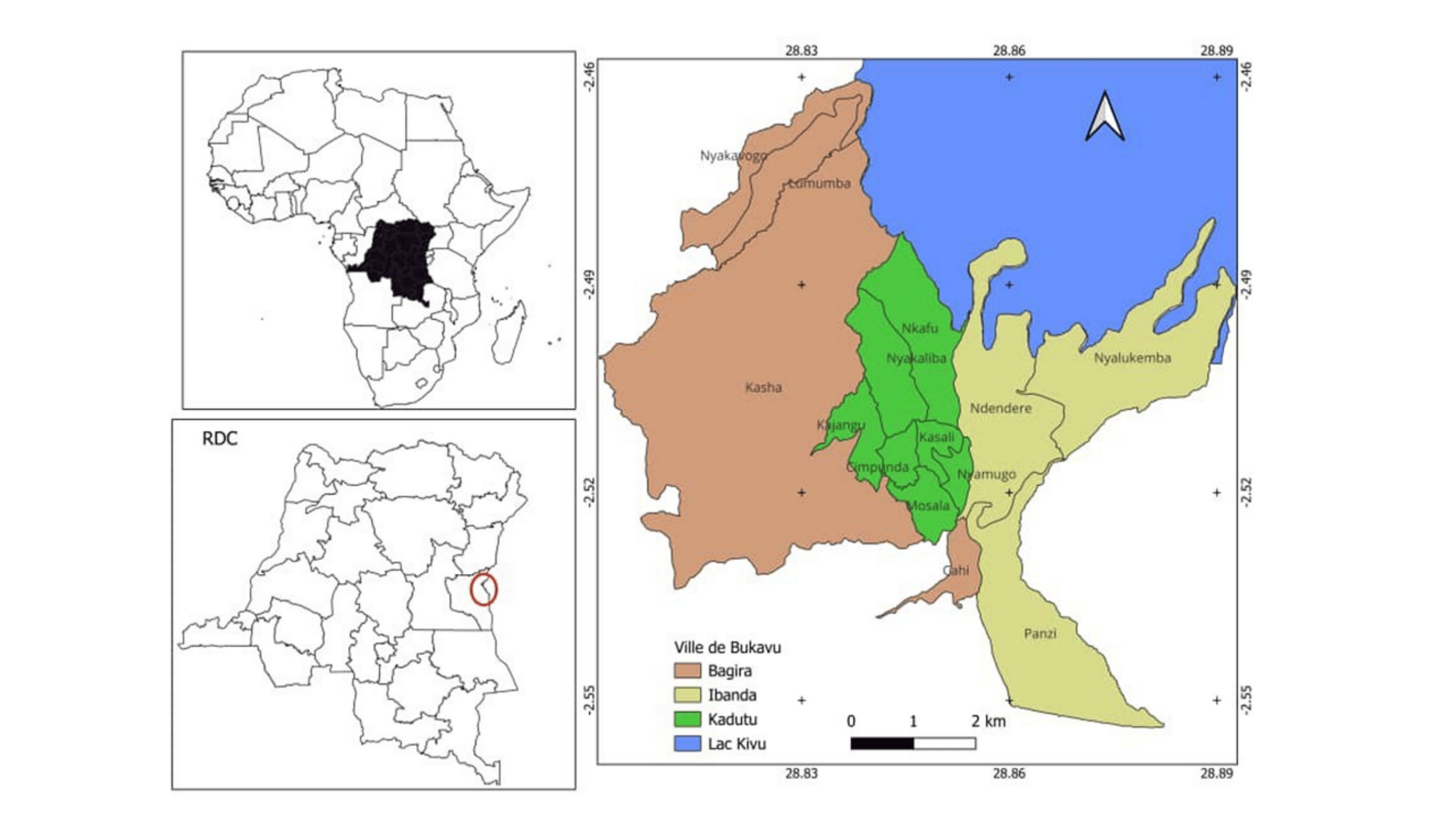
Map of Bukavu town highlighting the area covered by the study Bukavu town comprises three health zones: Bagira, Ibanda, and Kadutu. These zones are highlighted on the map using different colors. Image Credit: The image was created by the author using QGIS software (version 3.40.12; https://qgis.org/project/overview/).

The study included patients treated at Cliniques Universitaires de Bukavu, Skyborne Hospital, Clinique Saint-Luc Mbaki, and Centre Hospitalier Medicure in South Kivu, Democratic Republic of Congo (Figure 1).

Inclusion criteria comprised patients aged ≥17 years with distal radius fractures managed by percutaneous intrafocal pinning without an image intensifier, complete medical records, and a minimum follow-up of six months for clinical and radiological evaluation. Patients with incomplete records or follow-up less than six months were excluded.

Data collection and outcome assessment

Data were collected using a standardized form, extracting information from medical records, consultation registries, hospital databases, and operative reports. Fractures were classified using the modified Castaing classification as described by Kapandji. Postoperative outcomes were evaluated using the Grumillier criteria (subjective, objective, and radiological), as reported by the Groupe d’Étude de la Chirurgie Orthopédique (GECO) group.

Sample size

The study employed a consecutive sampling method, enrolling all eligible patients presenting to the emergency department of Cliniques Universitaires de Bukavu, Skyborne Hospital, Clinique Saint-Luc Mbaki, and Centre Hospitalier Medicure in South Kivu, between January 1, 2018, and December 31, 2023, resulting in a sample size of 101 participants, determined based on the study period and inclusion criteria.

Statistical analysis

Data were entered into Microsoft Excel (Microsoft Corporation, Redmond, WA, USA) and analyzed using R software (version 4.4.3, R Foundation for Statistical Computing, Vienna, Austria). Statistical analyses included descriptive statistics (mean, standard deviation, minimum, maximum) for frequency and outcome distribution. Contingency tables were examined using principal component analysis (PCA) and mixed-data factorial analysis to explore relationships between variables.

Surgical technique

The procedure was performed under general or locoregional anesthesia with the patient in dorsal decubitus and the upper limb positioned on an arm table, with or without a pneumatic tourniquet. No image intensifier was used. Fracture reduction was achieved through external maneuvers, including axial thumb traction, wrist flexion, and ulnar tilt. The fracture site was identified by digital palpation of the fragments using the operator’s thumb. Reduction quality was assessed by restoration of the bi-styloid line, radial anatomical axis, and correction of epiphyseal fragment deformity. Three percutaneous pins (18/10 or 20/10 mm, 30 cm long, cut into three segments) were inserted intrafocally: a dorsolateral pin along the second radius axis, a lateral pin along the first radius axis, and a dorsomedial pin along the third radius axis. Anatomical landmarks guided pin placement, with the lateral radial edge used for the lateral pin and the axes of the second and third for the dorsolateral and dorsomedial pins, respectively. Pins were cut flush and buried subcutaneously.

Postoperatively, an antebrachio-palmar cast was applied for 45 days. Pin removal occurred at 45 days. Patients initiated self-rehabilitation on postoperative day 1, performing isometric contractions of wrist and finger extensors/flexors and active finger mobilization. Physiotherapist-guided rehabilitation commenced after cast and pin removal to optimize functional recovery.

Ethical considerations

This study was approved by the Medical Ethics Committee of the Official University of Bukavu, Bukavu, Democratic Republic of Congo (Approval No. UOB/CEM/037/2023). Written informed consent was obtained from all participants for both treatment and the use of their data in this research, in accordance with the Declaration of Helsinki and institutional ethical guidelines.

## Results

The cohort included 101 patients with a mean age of 36.41 ± 12.61 years (range: 18-70 years). Males accounted for 68.32% (n = 69). Civil servants were the most affected occupational group (n = 37, 36.63%), followed by students (n = 13, 12.87%) and shopkeepers (n = 12, 11.88%). Urban residents comprised 78.22% (n = 79) of cases, with 19.80% (n = 20) from rural areas (Table 1).

**Table 1:** Socio-demographic characteristics of patients with distal radius fractures (n = 101) Of the 101 participants, 68.3% (n = 69) were male. Civil servants represented the largest occupational group (36.6%, n = 37), followed by students (12.9%, n = 13) and shopkeepers (11.9%, n = 12). Most participants were urban residents (78.2%, n = 79), with 19.8% (n = 20) from rural areas. ANR: Agence Nationale de Renseignements (National Intelligence Agency)

Characteristic	Value
Age (years)	
Minimum	18
Maximum	70
Mean ± SD	36.41 ± 12.61
Age range (years)	n (%)
18-27	31 (30.69)
28-37	23 (22.77)
38-47	27 (26.73)
48-57	13 (12.87)
58-67	6 (5.94)
68-77	1 (0.99)
Gender	
Male	69 (68.32)
Female	32 (31.68)
Profession	
Civil servant	37 (36.63)
Student	13 (12.87)
Retailer	12 (11.88)
Teacher	9 (8.91)
Housekeeper	9 (8.91)
Bricklayer	4 (3.96)
Player	4 (3.96)
Sports	4 (3.96)
Driver	3 (2.97)
Motorcyclist	1 (0.99)
ANR agent	1 (0.99)
None	4 (3.96)
Origin	
Urban	79 (78.22)
Rural	20 (19.80)
Foreign	2 (1.98)

Clinical and radiological characteristics

**Figure 2:**
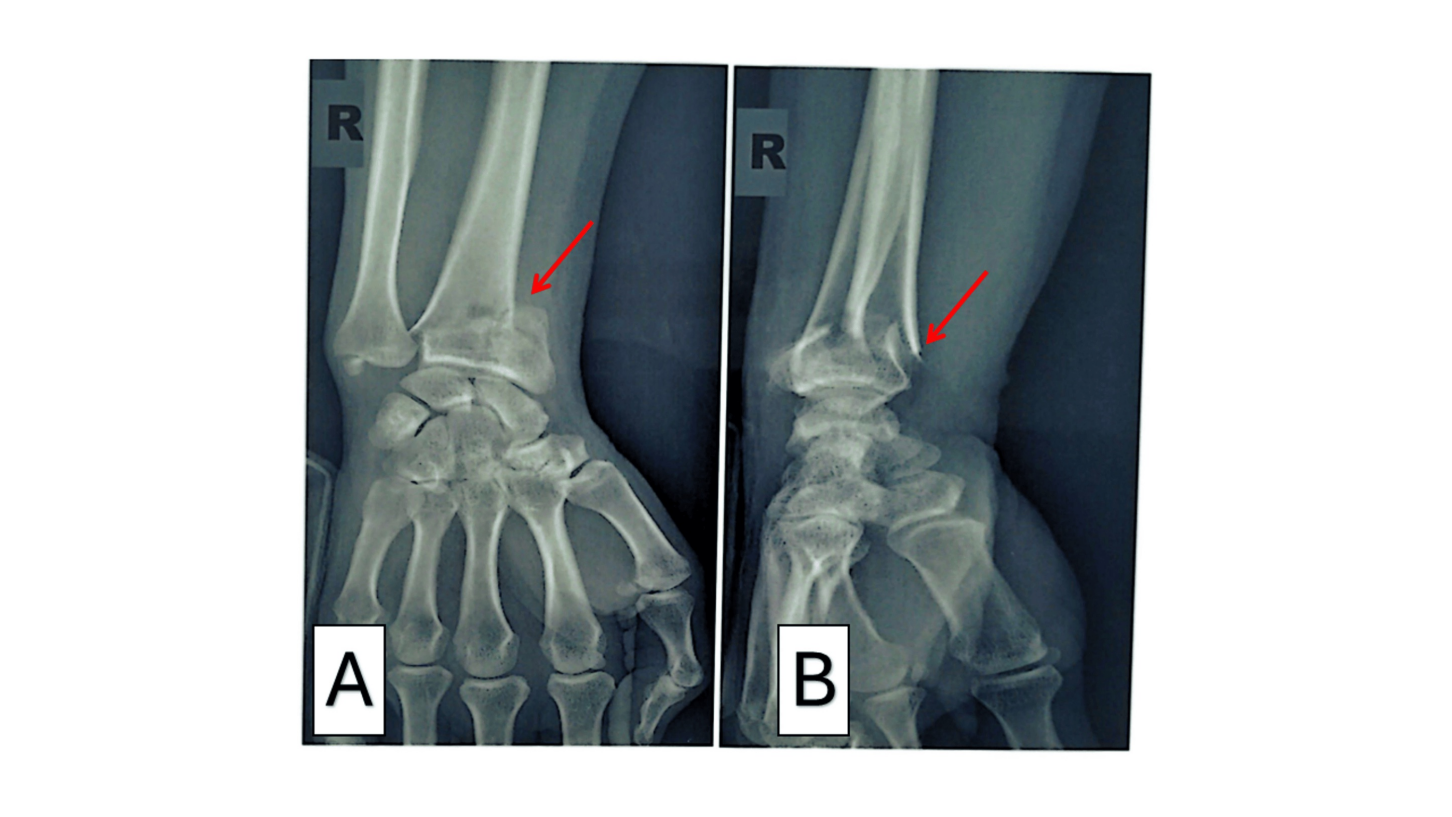
Anteroposterior (A) and lateral (B) radiographs of the forearm showing a distal radial fracture in a 33-year-old male patient following a fall A (anteroposterior view) and B (lateral view) demonstrate plain radiographs of the wrist. There is a compaction/impaction fracture of the distal radius characterized by cortical buckling and increased radiopacity (sclerosis) at the metaphyseal region, best appreciated on the lateral projection (Figure B, red arrow). Subtle disruption of the dorsal cortical margin with mild dorsal angulation of the distal fragment is noted, consistent with a compression-type distal radius fracture (Colles’ type pattern). No intra-articular extension seen; an associated ulnar styloid fracture is evident in the provided views.

Comorbidities were diabetes (n = 6, 5.94%) and osteoporosis (n = 3, 2.97%); 83.17% (n = 84) had no relevant medical history. Primary injury mechanisms were road traffic accidents (n = 36, 35.64%) and falls from height onto the hand (n = 32, 31.68%). The right wrist was involved in 53.47% (n = 54) and the left in 45.54% (n = 46). Per the modified Castaing classification, type I fractures (Figure 2) dominated (n = 93, 92.08%), followed by types 2 and 4 (n = 3 each, 2.97%) (Table 2).

**Table 2:** Clinical and radiological characteristics of patients with distal radius fractures (n = 101) Comorbidities were diabetes (n = 6, 5.94%) and osteoporosis (n = 3, 2.97%); 83.17% (n = 84) had no relevant medical history. Primary injury mechanisms were road traffic accidents (n = 36, 35.64%) and falls from height onto the hand (n = 32, 31.68%). The right wrist was involved in 53.47% (n = 54) and the left in 45.54% (n = 46). Per the modified Castaing classification, type I fractures dominated (n = 93, 92.08%), followed by types 2 and 4 (n = 3 each, 2.97%).

Characteristic	Category	n (%)
Mechanism of injury	Road traffic accident	36 (35.64)
	Fall from height	32 (31.68)
	Sports injury	16 (15.84)
	Fall from high place	6 (5.94)
	Domestic injury	3 (2.97)
	Occupational injury	3 (2.97)
	Slip	3 (2.97)
	Assault	1 (0.99)
	Other traffic injury	1 (0.99)
Affected side	Right	54 (53.47)
	Left	46 (45.54)
	Bilateral	1 (0.99)
Medical history	None	84 (83.17)
	Diabetes mellitus	6 (5.94)
	Osteoporosis	3 (2.97)
	Hypertension	2 (1.98)
	Osteoporosis and hypertension	2 (1.98)
	Osteoporosis, hypertension, sickle cell disease	2 (1.98)
	Menopause	1 (0.99)
	Osteoporosis, diabetes, hypertension	1 (0.99)
Castaing classification	Type I	93 (92.08)
	Type II	3 (2.97)
	Type III	1 (0.99)
	Type IV	3 (2.97)
	Type VIII	1 (0.99)

Perioperative details

**Figure 3:**
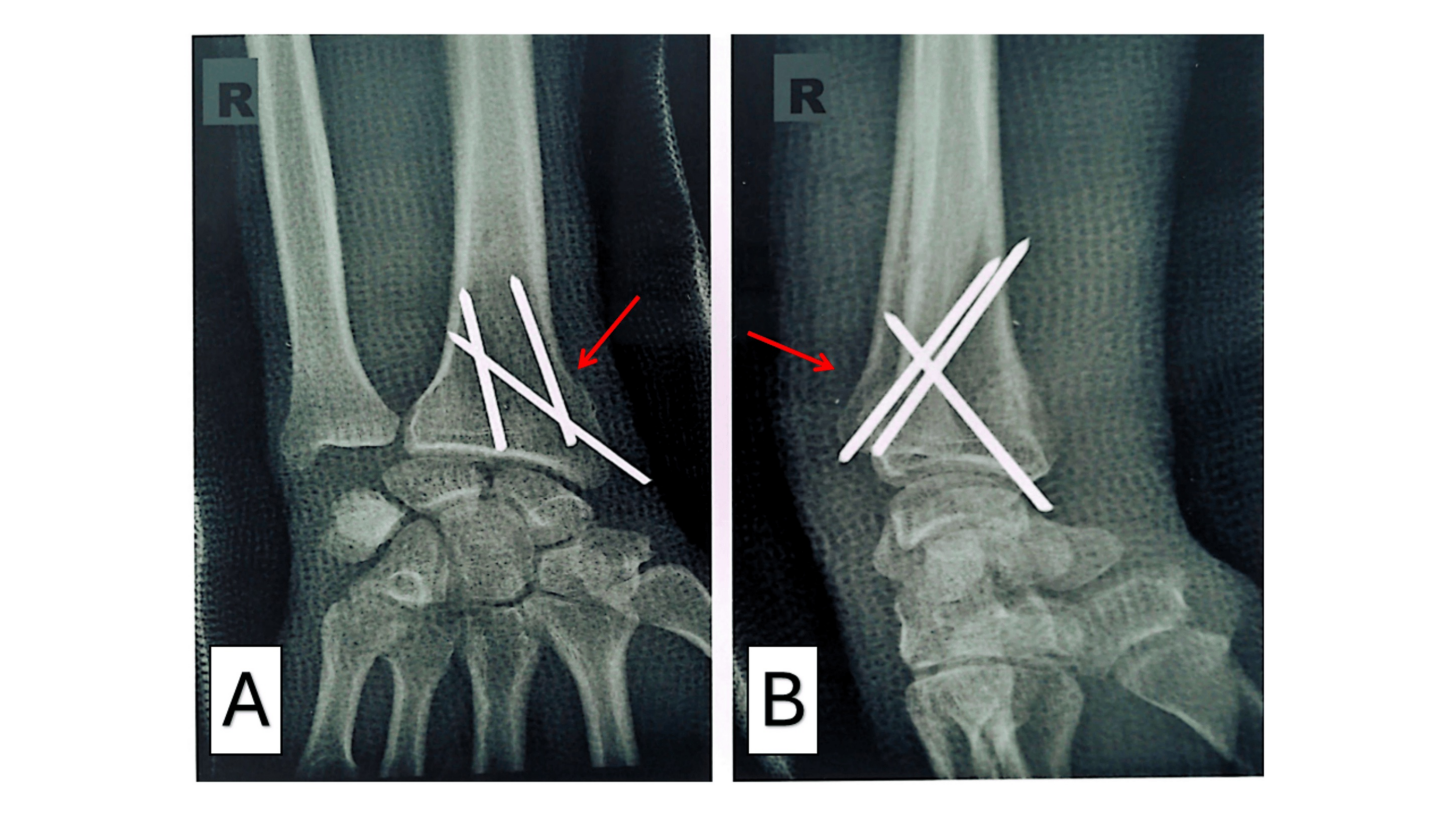
Postoperative anteroposterior (A) and lateral (B) radiographs showing reduction and osteosynthesis of the distal radius fracture using three pins placed with a freehand technique A (anteroposterior view) and B (lateral view) show postoperative plain radiographs of the wrist following closed reduction and percutaneous pinning of a distal radius compression fracture. The original injury was an extra-articular compaction/impaction fracture with dorsal cortical buckling and mild dorsal angulation of the distal fragment (Colles’ type pattern). Post-reduction alignment is restored with anatomical radial height, inclination, and near-neutral tilt. Fracture stability is achieved using three smooth percutaneous Kirschner wires (K-wires): Two parallel radial styloid pins were inserted from the dorsal-ulnar aspect of the styloid, engaging the opposite intact cortex (clearly visible in Figure A). One dorsoulnar pin crosses the fracture site obliquely, providing three-point fixation and buttressing the dorsal comminution (best seen in Figure B). The pins maintain fracture reduction without loss of position. No intra-articular penetration is observed. The configuration effectively neutralizes compressive and bending forces, ensuring stable fixation during healing.

**Figure 4:**
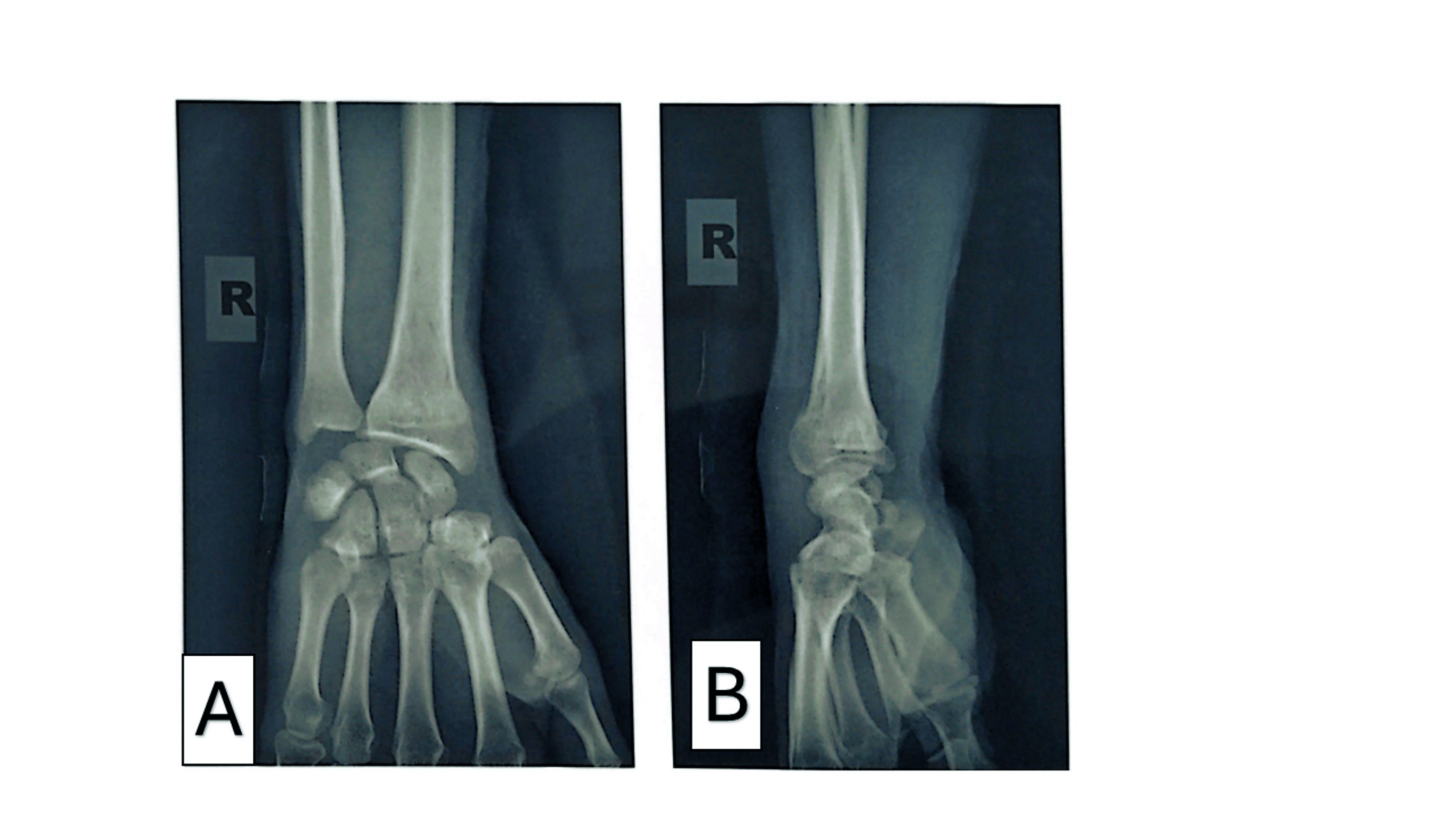
Radiograph of the same patient demonstrating a well-consolidated fracture 47 days after removal of the Kirschner wires (K-wires) A (anteroposterior view) and B (lateral view) are follow-up plain radiographs of the wrist obtained after removal of percutaneous K-wires, approximately seven weeks following closed reduction and pinning of an extra-articular distal radius compression fracture (Colles’ type). The fracture is fully consolidated with complete bony union across the metaphyseal region. Previous impaction and dorsal cortical buckling have remodeled, showing restoration of normal trabecular architecture and smooth cortical contours. Radial height, radial inclination, and volar tilt are anatomically restored with no residual angulation or displacement. The pin tracks are barely visible and show no signs of infection or osteolysis. There is no evidence of post-traumatic arthritis or secondary displacement. The radiographs confirm solid healing and excellent functional outcome.

All fractures underwent fluoroscopy-free percutaneous intrafocal pinning with three pins inserted via mini-incisions (posterolateral, lateral, and posteromedial, aligned with the second, first, and third ray axes, respectively) following reduction by external maneuvers (Figure 3). One patient required reintervention. The mean preoperative delay was 1.34 ± 0.52 days (range: 1-3 days), with 68.32% (n = 69) treated within 24 hours. General anesthesia and an arm tourniquet were universal, with patients positioned in dorsal decubitus. Postoperative radiographs (Figure 4) showed excellent alignment in 48.51% (n = 49), very good in 34.65% (n = 35), and good in 12.87% (n = 13). All patients received an antebrachio-palmar cast for 45 days (Table 3).

**Table 3:** Perioperative and postoperative characteristics of patients with distal radius fractures (n = 101)

Characteristic	Category	n (%)
Preoperative delay (days)	1	69 (68.32)
	2	30 (29.70)
	3	2 (1.98)
	Mean ± SD	1.34 ± 0.52
	Range	1-3
Surgical technique	External reduction	101 (100.00)
	Arm tourniquet	101 (100.00)
	Percutaneous intrafocal pinning	101 (100.00)
	Number of pins: Three	101 (100.00)
	Image intensifier: None	101 (100.00)
Postoperative immobilization	Immobilization: Yes	101 (100.00)
	Type: Antebrachio-palmar cast	101 (100.00)
	Type: Brachio-antebrachio-palmar cast	0 (0.00)
Postoperative radiographic results	Excellent	49 (48.51)
	Very good	35 (34.65)
	Good	13 (12.87)
	Fair	3 (2.97)
	Poor (required surgical revision)	1 (0.99)

Postoperative outcomes and complications

Mean hospital stay was 3.70 ± 1.12 days (range: 1-7 days), with 48.51% (n = 49) discharged after three days. Fracture consolidation occurred at a mean of 46.82 ± 4.67 days (range: 44-60 days), with 73.27% (n = 74) consolidating by 45 days. Pins and casts were removed at 50.67 ± 6.01 days (range: 45-60 days). Rehabilitation averaged 17.72 ± 3.22 sessions (range: 10-30), with 23.76% (n = 24) requiring 15 sessions. Complications included superficial infections (n = 3, 2.97%), malunion (n = 3, 2.97%), pin migration (n = 2, 1.98%), complex regional pain syndrome (n = 1, 0.99%), twisted pin (n = 1, 0.99%), radial sensory nerve injury (n = 1, 0.99%), and extensor pollicis longus tendon injury (n = 1, 0.99%) (Table 4).

**Table 4:** Postoperative course characteristics of patients with distal radius fractures (n = 101) The mean duration of hospital stay was 3.70 ± 1.12 days (range 1-7 days), with nearly half of the patients (48.51%, n = 49) discharged on the third postoperative day. Radiographic consolidation was achieved at a mean of 46.82 ± 4.67 days (range 44-60 days). The majority of fractures (73.27%, n = 74) consolidated at exactly 45 days, and 90.1% of cases showed union by 50 days. Pins and plaster cast were removed at a mean of 50.67 ± 6.01 days (range 45-60 days). Patients were followed up for a median of 24 months (mean 27.8 months, range 9-53 months), with the largest proportion (17.82%, n = 18) completing a 24-month follow-up.

Characteristic	Category	n (%) or Value
Length of hospital stay (days)	1	1 (0.99)
	2	6 (5.94)
	3	49 (48.51)
	4	19 (18.81)
	5	19 (18.81)
	6	6 (5.94)
	7	1 (0.99)
	Mean ± SD	3.70 ± 1.12
	Range	1-7
Time to consolidation (days)	44	3 (2.97)
	45	74 (73.27)
	46	6 (5.94)
	47	1 (0.99)
	48	3 (2.97)
	49	1 (0.99)
	50	3 (2.97)
	60	10 (9.90)
	Mean ± SD	46.82 ± 4.67
	Range	44-60
Time to removal of pins and cast (days)	Mean ± SD	50.67 ± 6.01
	Range	45-60
Last postoperative follow-up (months)	9	7 (6.93)
	10	1 (0.99)
	12	9 (8.91)
	16	4 (3.96)
	18	9 (8.91)
	20	1 (0.99)
	22	2 (1.98)
	24	18 (17.82)
	28	6 (5.94)
	30	8 (7.92)
	32	9 (8.91)
	36	11 (10.89)
	39	1 (0.99)
	46	4 (3.96)
	48	8 (7.92)
	49	1 (0.99)
	50	1 (0.99)
	53	1 (0.99)
	Mean ± SD	27.27 ± 11.85
	Range	9-53

Functional and radiological results

At a mean follow-up of 27.27 ± 11.85 months (range: 9-53 months), Grumillier criteria outcomes were subjective scores - very good in 84.16% (n = 85) and good in 9.90% (n = 10); objective scores - very good in 77.23% (n = 78) and good in 16.83% (n = 17); and radiological scores (Castaing) - very good in 75.25% (n = 76) and good in 19.80% (n = 20) (Table 5).

**Table 5:** Postoperative outcomes of patients with distal radius fractures (n = 101) Postoperative complications were uncommon (11.9%). Superficial pin-site infection and malunion each occurred in three patients (3.0%), followed by pin migration and complex regional pain syndrome in two patients each (2.0%). One case each of twisted pin, radial sensory nerve neurapraxia, and extensor pollicis longus irritation was recorded. There were no deep infections, nonunions, delayed unions, or post-traumatic osteoarthritis. K-wires and cast were removed at a mean of 50.7 ± 6.0 days (range 45-60 days). Patients underwent a mean of 17.7 ± 3.2 physiotherapy sessions. Functional and radiological outcomes were excellent or good in the vast majority: subjective score very good/good in 94.1%, objective score in 94.1%, and Castaing radiological score in 95.0%. Full socio-professional reintegration was achieved in 90.1% of patients.

Characteristic	Category	n (%) or Value
Postoperative complications	Superficial infection	3 (2.97)
	Pin migration	2 (1.98)
	Complex regional pain syndrome	2 (1.98)
	Malunion	3 (2.97)
	Twisted pin	1 (0.99)
	Radial sensory nerve injury	1 (0.99)
	Extensor pollicis longus tendon injury	1 (0.99)
	Wounds	0 (0.00)
	Delayed consolidation	0 (0.00)
	Nonunion	0 (0.00)
	Osteoarthritis	0 (0.00)
Rehabilitation sessions	10	1 (0.99)
	13	1 (0.99)
	14	3 (2.97)
	15	24 (23.76)
	16	15 (14.85)
	17	8 (7.92)
	18	18 (17.82)
	19	3 (2.97)
	20	17 (16.83)
	21	2 (1.98)
	22	3 (2.97)
	25	4 (3.96)
	30	2 (1.98)
	Mean ± SD	17.72 ± 3.22
	Range	10–30
Removal of pins and cast (days)	45	26 (25.74)
	46	8 (7.92)
	47	4 (3.96)
	48	17 (16.83)
	49	4 (3.96)
	50	15 (14.85)
	52	1 (0.99)
	55	1 (0.99)
	59	1 (0.99)
	60	24 (23.76)
	Mean ± SD	50.67 ± 6.01
	Range	45–60
Outcome scores		
Subjective score	Very good	85 (84.16)
	Good	10 (9.90)
	Fair	6 (5.94)
Objective score	Very good	78 (77.23)
	Good	17 (16.83)
	Fair	4 (3.96)
	Poor	2 (1.98)
Radiological score (Castaing)	Very good	76 (75.25)
	Good	20 (19.80)
	Fair	5 (4.95)
Socio-professional reintegration	Yes	91 (90.10)
	No	1 (0.99)
	Unemployed/retired	9 (8.91)

Statistical insights

Principal component analysis (PCA) indicated that prolonged rehabilitation correlated with worse subjective and objective scores, while better scores showed a negative correlation. Castaing fracture types were inversely correlated with outcomes, with type I fractures achieving superior results compared to types 4 and 8. Extended hospital stays were associated with poorer objective scores and inversely related to better scores. Factorial analysis with mixed data confirmed that poor objective scores predicted failed socio-professional reintegration. Age and gender did not influence outcomes across subjective, objective, or radiological domains.

## Discussion

This study demonstrates the efficacy of fluoroscopy-free percutaneous intrafocal pinning for distal radius fractures in a resource-limited setting, yielding excellent anatomical, radiological, and functional outcomes. Among 101 patients, predominantly with Castaing type I fractures (92.08%), very good subjective (84.16%), objective (77.23%), and radiological (75.25%) results were achieved at a mean follow-up of 27.27 months. Complications were minimal, and early rehabilitation contributed to robust recovery. These findings highlight the technique’s potential as a safe, effective alternative in environments lacking advanced imaging, challenging the reliance on fluoroscopy while affirming its applicability in low-resource contexts.

Epidemiological profile

In this series, males predominated (68.32%, n = 69), consistent with findings from Traore et al. and Parupalli and Mithun [16,17], but contrasting with studies reporting female predominance [18-20]. The male predominance likely reflects the higher exposure of young men to high-risk occupations and activities in South Kivu, such as manual labor and transportation. The mean age was 36.41 ± 12.61 years (range: 18-70), aligning with African series [17,21] but younger than Western cohorts [4]. This younger age profile may be attributed to the high-energy trauma mechanisms prevalent in a youthful Congolese population. Civil servants (n = 37, 36.63%), students (n = 13, 12.87%), and shopkeepers (n = 12, 11.88%) were the most affected occupations, mirroring patterns in African studies [16,17]. These groups’ active and mobile lifestyles increase their exposure to road traffic accidents and falls, the leading causes of injury in this study. Urban patients (78.22%, n = 79) outnumbered rural ones (19.80%, n = 20), differing from Dworkin et al. [22], likely due to the urban location of study hospitals facilitating easier access. Diabetes (n = 6, 5.94%) and osteoporosis (n = 3, 2.97%) were the primary comorbidities, consistent with Nemmar et al. [23]. These conditions may exacerbate bone fragility, contributing to fracture risk.

Clinical characteristics

Road traffic accidents (35.64%, n = 36) and falls from height (31.68%, n = 32) were the predominant etiologies, aligning with Traore et al. and Parupalli and Mithun [16,17]. This reflects South Kivu’s poor road infrastructure, non-compliance with traffic regulations, and mountainous terrain, which predispose to falls. The right wrist was more commonly affected (53.47%, n = 54) than the left (45.54%, n = 46), consistent with Kacimi et al. [18], likely due to the dominance of the right hand in most individuals, though some studies (e.g., Nemmar et al., 2024) [23] report otherwise, suggesting variability by chance or population.

Radiological findings

Per the modified Castaing classification, type I fractures dominated (92.08%, n = 93), followed by types 2 and 4 (2.97%, n = 3 each). The prevalence of extra-articular fractures, particularly Pouteau-Colles type, aligns with multiple studies [17,18,24,25]. This pattern likely results from the common compression-extension mechanism in this cohort, reflecting the high-energy trauma observed.

Treatment and perioperative outcomes

All fractures were reduced using external maneuvers and stabilized with fluoroscopy-free percutaneous intrafocal pinning, employing three pins via mini-incisions. Immediate postoperative radiographs showed excellent (48.51%, n = 49), very good (34.65%, n = 35), or good (12.87%, n = 13) alignment, underscoring the technique’s efficacy despite the absence of intraoperative imaging. The mean preoperative delay was short (1.34 ± 0.52 days), comparable to Ayouba et al. [15] and Panthi et al. [19], facilitated by urban proximity to hospitals and the urgency of pain-driven consultations. General anesthesia was universal, reflecting limited resources for locoregional anesthesia, consistent with Chgoura [25]. The mean hospital stay (3.70 ± 1.12 days) was slightly longer than reported by Panthi et al. [19] and Kacimi et al. [18], possibly due to socioeconomic constraints or insurance coverage variations. Consolidation occurred at 46.82 ± 4.67 days, within the expected four- to six-week range, similar to Ayouba et al. [15] and Khadka et al. [24], likely due to precise reduction and stable fixation. Pin and cast removal averaged 50.67 ± 6.01 days, aligning with standard protocols.

Complications and rehabilitation

Complications were infrequent, including superficial infections (2.97%, n = 3), malunion (2.97%, n = 3), pin migration (1.98%, n = 2), complex regional pain syndrome (0.99%, n = 1), twisted pin (0.99%, n = 1), radial sensory nerve injury (0.99%, n = 1), and extensor pollicis longus tendon injury (0.99%, n = 1). These rates are lower than or comparable to other series [22,25], likely due to meticulous surgical technique, including short incisions and early rehabilitation. Rehabilitation averaged 17.72 ± 3.22 sessions, consistent with Ayouba et al. [15], emphasizing its role in recovery. Peyroux et al. highlight physiotherapy’s benefits in reducing pain, edema, and improving function, supporting its critical role in this cohort [26].

Functional and radiological outcomes

At a mean follow-up of 27.27 ± 11.85 months, outcomes were excellent: subjective scores were very good in 84.16% (n = 85) and good in 9.90% (n = 10); objective scores were very good in 77.23% (n = 78) and good in 16.83% (n = 17); and radiological scores were very good in 75.25% (n = 76) and good in 19.80% (n = 20). These results align with Peyroux et al. [26], Nemmar et al. [23], and Parupalli and Mithun [17], surpassing some series (e.g., Ayouba et al. [15]). The consistency between functional and radiological outcomes reflects the young cohort’s recovery potential, precise surgical technique, and early rehabilitation. Notably, subjective and objective functional results slightly outperformed radiological outcomes, possibly due to the adaptive capacity of younger patients.

Statistical correlations

Principal component analysis (PCA) revealed that prolonged rehabilitation correlated with poorer scores, while shorter rehabilitation predicted better outcomes. Castaing type I fractures yielded superior results compared to types 4 and 8, reflecting simpler fracture patterns. Longer hospital stays were associated with worse objective scores, likely indicating more complex cases. Factorial analysis confirmed that poor objective scores predicted failed socio-professional reintegration, underscoring the societal impact of functional outcomes. Age and gender had no influence on outcomes, consistent with the literature.

Implications of fluoroscopy-free technique

The absence of an image intensifier, a potential limitation, did not compromise outcomes, challenging reliance on intraoperative imaging in resource-limited settings. While fluoroscopy ensures precision, its risks, including radiation-induced skin, thyroid, and ocular damage [12,13], highlight the value of alternative approaches. This study supports fluoroscopy-free pinning as a viable, safe technique, particularly where resources are scarce, echoing Nordback et al. [7].

Limitations

This study has several limitations. First, the absence of intraoperative fluoroscopy may have reduced the precision of fracture reduction and pin placement, particularly for complex fracture patterns, although outcomes remained favorable. Second, the cohort was predominantly composed of Castaing type I fractures (92.08%), potentially limiting the generalizability of fluoroscopy-free percutaneous intrafocal pinning to more complex distal radius fractures. Third, the study did not include a control group treated with fluoroscopy-guided pinning, precluding direct comparison of efficacy and safety between techniques. Fourth, long-term complications beyond the mean follow-up of 27.27 months were not assessed, possibly underestimating late sequelae specific to this approach. Finally, the reliance on manual reduction and anatomical landmarks introduces operator-dependent variability, which may affect reproducibility in less experienced hands.

## Conclusions

Fluoroscopy-free percutaneous intrafocal pinning proved to be a highly effective and safe technique for managing distal radius fractures in adults in the resource-constrained setting of South Kivu, Democratic Republic of Congo. This prospective study demonstrated excellent anatomical, radiological, and functional outcomes, with very good subjective, objective, and radiological results at a mean follow-up period. The low complication rate and rapid consolidation underscore the technique’s reliability, even without intraoperative imaging. These findings challenge the necessity of fluoroscopy in simpler fracture patterns, such as Castaing type I, and highlight the critical role of early rehabilitation in optimizing recovery. By offering a viable alternative to resource-intensive methods, this approach holds significant promise for improving fracture care in low-resource settings globally. Future research should focus on comparative studies with fluoroscopy-guided pinning and long-term outcomes across diverse fracture types to further validate and refine this technique.
